# Sarcoma Stem Cells: Do We Know What We Are Looking for?

**DOI:** 10.1155/2012/291705

**Published:** 2012-05-09

**Authors:** Matteo Trucco, David Loeb

**Affiliations:** Division of Pediatric Oncology, Department of Oncology, Sidney Kimmel Comprehensive Cancer Center, Johns Hopkins University, Bunting-Blaustein Cancer Research Building, Room 2M51, 1650 Orleans Street, Baltimore, MD 21231, USA

## Abstract

Sarcomas represent a heterogeneous group of cancers thought to originate from malignant transformation of mesenchymal cells. There is increasing evidence that many, if not all, sarcomas contain within them tumor-initiating, or “cancer stem,” cells responsible for the initiation, maintenance, and potentially relapse and metastasis of the tumor. Various techniques have been adopted in recent years to identify putative sarcoma stem cell populations. The goal of this paper is to summarize the criteria used to identify a stem cell population, describe the more prominent markers and techniques used to isolate cancer stem cells in sarcomas, and review the evidence for the existence of cancer stem cells in sarcomas.

## 1. Introduction

Sarcomas represent a heterogeneous group of cancers that seem to originate from malignant transformation of mesenchymal cells. Whether they represent malignant transformation of mesenchymal stem cells or of differentiated cells of mesenchymal origin has not been established. There is increasing evidence that many, if not all, cancers constitute a hierarchy of cells including so-called cancer stem cells that are believed to be the progenitor cells from which the tumor was spawned and that these cancer stem cells may be responsible for relapses and metastases [1]. Cancer stem cells appear to be resistant to chemotherapy, may remain quiescent for extended periods, perhaps have an affinity for hypoxic environments, and may have a predisposition for migration and metastasis. Additionally, since the cancer stem cell model suggests that these cells make up a very small portion of the tumor bulk, the majority being their progeny, the model also would offer an explanation for relapses that occur despite what would appear to be a total response to initial therapeutic interventions.

An underlying matter is the origin of cancer stem cells. The use of the term “cancer stem cells” is suggestive that these are normal stem cells that have undergone malignant transformation and thus have become cancerous. Though this interpretation is understandable and this is a plausible source of cancer stem cells, it is by no means established that this is how cancer stem cells come to be. Alternatively, cancer stem cells may derive from differentiated cells that, through malignant transformation, acquire properties reminiscent of stem cells. To avoid the ambiguity of the term “cancer stem cell,” for the remainder of this review the terms tumor-initiating cell (TIC) and sarcoma-initiating cell (SIC) will be used.

To better study SICs and develop therapies to target them, they must first be identified and isolated. Several techniques for identifying and isolating TIC have been used with varying success in other more common malignancies, and these techniques are being studied on the gamut of sarcomas. Most of these techniques involve identifying a subpopulation of cancer cells that have properties typically seen only in normal stem cells. We will describe the more prominent techniques for TIC identification and isolation being used in sarcomas (Table 1) and discuss the evidence supporting the existence of SICs.

## 2. Stem Cell Assays

The definition of a TIC and the means by which to determine that a population contains TICs remain points of contention [2]. The following are the most commonly used assays for stem cell-like properties. They include functional tests of the ability to behave like a TIC as well as descriptive assays evaluating if cells have qualities expected in TICs. 

### 2.1. Functional Assays

#### 2.1.1. Clonogenic/Sphere-Forming Assay

TICs are believed to have an increased ability to form colonies from a single cell as compared to their progeny [3]. Colony-forming assays are performed by preparing a single cell suspension and plating the cells in soft agar. Colonies that grow from the individual cells are typically stained with crystal violet and counted and measured using a stereomicroscope. The ability to form colonies in soft agar is presumed to be unique to stem cells, but one may question if a malignant cell line able to grow *in vitro* may not also be capable of growing in soft agar. Another issue with this assay is whether the size of the colony is a reflection of “stemness” (typically smaller colonies are excluded from the counts). The size of the colony may simply be a measure of proliferation or quiescence and not presence or absence of a TIC. Additionally, clonogenic assay results can be influenced by technical considerations. The agar is typically autoclaved and mixed with media containing sufficiently diluted cells when the temperature is low enough to not kill the cells, but still warm enough to be poured into wells. Proper cell dilution is critical to ensure that each colony results from a single cell, and toxicity to cells from agar that is too hot may also affect results.

Sphere-forming assays assess the ability of cells to grow as spheres in nonadherent conditions [4]. This is also thought to be a unique property of TICs, but some cell lines appear to display this ability ubiquitously. The same issues of cell dilution apply to this assay, as cell clumps bear a striking resemblance to spheres. Finally, what both of these assays really measure is a cell's ability to behave like a TIC when removed from an *in vivo* niche, rather than truly detecting TICs.

#### 2.1.2. Tumorigenesis

The ability to grow serially transplantable tumors in immunodeficient mice is considered by some to be the “gold standard” characteristic of TICs [5]. Theoretically, a single TIC should be able to generate a tumor; however, no studies have been able to purify a population to that degree. What usually is reported is that the putative TIC population is able to grow a tumor with a smaller number of cells inoculated into a mouse than the nonTIC population. There are, however, no set criteria for what is considered a significant difference between tumorigenesis in the two populations, and there is no consensus on what strain of mouse to test tumorigenesis.

#### 2.1.3. Dye Efflux

ATP-binding cassette (ABC) transporter efflux of DNA-binding dyes such as Hoechst 33342 or Rhodamine 123 is also used as an indicator of stem cell properties [6]. Exclusion of the dye is what identifies “side population” (SP) by flow cytometry. The activity of the ABC transporters can be blocked by the calcium-channel blocker verapamil. One of the challenges of using SP as a measure of TICs is that Hoechst dye, for example, may be toxic to cells [7]. As such, cells that are identified by exclusion of this dye will have a survival advantage over cells that are not able to efflux the dye as readily. There is also evidence that TICs can be found in the nonSP, suggesting that exclusion of Hoechst 33342 is not an essential quality of TICs [8, 9].

#### 2.1.4. Chemoresistance

Somewhat related to drug efflux, chemotherapy resistance is also considered a hallmark of TICs [10]. Part of what makes the cancer stem cell theory attractive is the idea that the TICs survive all therapeutic interventions and then may cause relapse and metastases [11]. Thus, dose response curves to standard chemotherapeutic agents are performed on candidate TIC populations and compared to the nonTIC population. It is expected that the TICs will demonstrate relative resistance to chemotherapy. One potential confounding factor is that TICs may avoid the toxicity of chemotherapy by remaining quiescent, since chemotherapy tends to be more toxic to rapidly proliferating cells. If this is the case, a proliferation or viability assay may underestimate the resistance of the TICs if their resistance is based on quiescence rather than expression of resistance-associated proteins like aldehyde dehydrogenase or ABC transporters. Additionally, what degree of change in the IC_50_ that constitutes “chemoresistance” has not been established.

#### 2.1.5. Differentiation

A suspected TIC's ability to differentiate along a variety of lineages is considered an indicator of pluripotency [12]. In sarcoma research, differentiation into mesenchymal cell types such as osteoblasts, adipocytes, and chondrocytes is typically evaluated, based on the theory that a SIC is a mesenchymal stem cell that has undergone malignant transformation. If, however, a SIC is a fully differentiated cell that has acquired stem-like properties, it may not be capable of transdifferentiation to a different cell type. Furthermore, differentiating media may induce differentiation in cancer cells regardless of their “stemness.”

### 2.2. Descriptive Assays

#### 2.2.1. Expression of Stem Cell Genes

Expression of so-called “stem cell genes” such as OCT4, SOX2, and NANOG is also used as a marker of TICs. These genes are transcription factors found in embryonic stem cells and appear to be necessary for maintenance of an undifferentiated state, pluripotency, and self-renewal [13]. However, there are many such genes, and the number necessary to confer a “stem cell” designation, as well as the level of expression necessary, remains unclear. Moreover, these genes are often expressed not only in stem cells but also in certain committed progenitors, calling into question their specificity as “stem cell genes.” Expression of these genes may simply be a function of the malignant transformation of the cells and not unique to a TIC subpopulation.

#### 2.2.2. Aldehyde Dehydrogenase Activity

Hematopoetic and neural stem/progenitor cells have been shown to have high activity of aldehyde dehydrogenase (ADLH) [14, 15]. It is thus hypothesized that TICs would also have higher levels of ALDH activity. ALDH “bright” or “high” cells are typically isolated by incubating cells with a fluorescent reagent (e.g., ALDEFLUOR), which is activated by ALDH. Flow cytometry is then used to identify and compare the amount of ALDH activity in a putative TIC population as compared to nonTIC population.

## 3. Approaches to Stem Cell Isolation

### 3.1. Cell Surface Markers

 The first definitive work describing TIC was performed in Acute Myeloid Leukemia where the surface marker phenotype CD34+, CD38− identifies a small subpopulation of cells that is able to propagate the leukemia, while CD34+ cells that express CD38 cannot, despite having the morphologic phenotype of leukemic blasts [16]. These findings prompted other researchers to use surface markers to identify TIC in other malignancies including breast, brain, lung, prostate, melanoma, pancreas, colon, liver, head and neck, and ovarian cancers [17]. Several surface markers have been studied in multiple malignancies as identifiers of TIC including CD20, CD24, CD34, CD44, CD90, CD117, and CD133 [17].

 Of these, CD133 has garnered the most attention in sarcomas. Originally described in 1997, CD133 is a 5-transmembrane protein whose expression was restricted to CD34^bright^ cells suggesting it was a viable marker of hematopoetic stem and progenitor cells [18]. It was subsequently identified in epithelial cells and in endothelial and neural progenitor cells. It was also used to identify potential TIC in various cancers, including brain, colon, liver, lung, ovarian, pancreatic, and prostate [17]. CD133's function and/or role in pluripotency remains unclear. CD133 appears to be concentrated in plasma membrane protrusions such as microvilli [19]. In addition, frameshift mutations of CD133 have been associated with retinal degeneration [20]. The relevance of these findings to stem cell biology, however, remains unclear.

The biological function of the surface markers used to identify TIC, such as CD133, is often unclear. Though our lack of understanding does not negate usefulness as a cancer stem cell marker, furthering our knowledge about these markers could help clarify their importance as markers of stem cells and may lead to discovery of more accurate markers (e.g., downstream targets). Furthermore, if a select surface marker is simply a marker of cell viability, differences between clonogenicity, sphere formation, tumorigenicity, and so forth, may reflect a comparison between viable and nonviable cells instead of stem cells and nonstem cells.

Interestingly, CD133 has been detected on the surface of differentiated epithelial cells in a variety of tissues, and at least one study has shown that CD133 expression is not limited to tumor-initiating cells in colon cancer [33, 34]. In addition, some studies have demonstrated tumor-initiating activity in cells that are CD133 negative. Finally, other studies have compared CD133 with other presumed markers of TIC. Though the CD133+ cells still showed stem-like properties, the CD133 cells are often outperformed in assays of cancer stem cell function by cells identified by other techniques, such as ALDH activity [35, 36]. 

### 3.2. ALDH

Aldehyde dehydrogenase (ALDH) is a detoxifying enzyme responsible for oxidation of intracellular aldehydes [37]. It is found in many cells throughout the body, but hematopoietic and neural stem/progenitor cells have particularly high ADLH activity [38–40]. ALDH may play a role in early differentiation of stem cells through oxidizing retinol to retinoic acid and can confer resistance to chemotherapeutic agents such as cyclophosphamide [41, 42]. High ALDH activity has been demonstrated in TIC populations of breast, colon, and lung cancers among others [1]. Just as high ALDH activity can be used as a measure of “stemness,” isolation of the population with the highest level of ALDH activity is thought to enrich for TICs. Typically, cells with the highest ALDH activity, “ALDH-high”, are compared to the subpopulation that shows the lowest ALDH activity, “ALDH-low”, as well as cells that are simply passed through the flow cytometer without ALDH selection, “flow through”.

One of the biggest challenges of using ALDH as a marker of TIC is the arbitrary nature of the 2 or 3% cut-off of cells with the highest and lowest ALDH activity. Additionally, ALDH is a marker of cell viability or detoxifying ability, so perhaps the enhanced ability of ALDH-high cells to grow colonies, spheres, or *in vivo* tumors may simply reflect a healthier cell population.

### 3.3. Side Population

Goodell et al. noted a distinct population of murine bone marrow cells that stained poorly with the Hoechst 33342 dye [43]. Cells within this population, termed the “side population” (SP) due to its position in the flow cytometry plot, were found to have many of the characteristics of hematopoetic stem cells. This group determined that the reason for the low Hoechst staining was efflux of the dye mediated by ATP-binding cassette (ABC) transporter proteins. Chemotherapy drugs are also substrates for these efflux pumps, suggesting a mechanism for drug resistance in these cells. SPs have since been used to identify both normal stem cell and TIC populations. Several groups have used SP as a sign of “stemness” to support the idea that the marker they were studying identified stem cells; however, identifying a putative cancer stem cell population by isolating the SP has a few shortcomings including inherent toxicity of the dye giving cells that efflux it a survival advantage and that stem cells have been isolated from the nonSP as well, suggesting that dye efflux is not an essential characteristic of stem cells [17–19].

## 4. Evidence for TIC in Sarcomas

 The assays discussed above have been applied to numerous sarcoma subtypes (Table 2). This work has been variably successful and is summarized below.

### 4.1. Osteosarcoma

 Tirino et al. isolated a CD133+ subpopulation in osteosarcoma cell lines SAOS2, MG63, and U2OS that demonstrated increased stem-like properties as compared to the CD133− population [21]. Their experiments included sphere formation, growth in soft agar, expression of the stem cell gene OCT3/4, presence of a side-population as evidenced by Hoechst dye exclusion, and growth and proliferation assays. Subsequent flow cytometric analysis of 21 human primary sarcomas and 2 osteosarcoma cell lines derived from biopsies again demonstrated that CD133+ cells exhibit stem cell-like properties [22].

Adhikari et al. investigated a population that was CD117 and Stro-1 double-positive as a potential cancer stem cell in osteosarcoma [23]. CD117, also known as c-KIT, is a protooncogene that is expressed on many hematopoietic progenitor cells. Stro-1 is believed to be a marker of osteogenic progenitor cells in bone marrow. Both murine and human osteosarcoma cells were plated in a sphere-forming assay, and the cells from the spheres were then analyzed for expression of stem cell markers compared to the same cell lines growing in a monolayer. CD117, Stro-1, and the ABC transporter ABCG2 were expressed at higher levels in the sphere cells. The sphere cells from mouse osteosarcoma cell lines were more tumorigenic, with 2 of 5 and 2 of 4 mice growing tumors with as few as 200 cells isolated from spheres injected. Double-positive (DP) cells (CD117+ and Stro-1+) from 3 mouse osteosarcoma cell lines were also more tumorigenic with 7 of 7 and 4 of 6 mice growing tumor when injected with 200 DP cells with two of the cell lines and 3 of 5 mice growing tumor with 2,000 DP cells injected from a third mouse osteosarcoma cell line. None of the mice injected with comparable double-negative (DN) cells from either cell line grew tumor. Comparing DP to DN cells from three human osteosarcoma cell lines showed 7 of 8 versus 1 of 8 mice growing tumor when injected with equal number of DP or DN cells, respectively, in two of the cell lines, while the third cell line showed that 3 of 4 mice injected with DP cells grew tumor compared to 0 of 4 injected with the same number of DN cells from the same human osteosarcoma cell line. Finally, comparison between DP and DN cells from two of the mouse osteosarcoma cell lines showed a shift in IC_50_ and increased cell survival percentage in the DP cells when treated with doxorubicin.

Wang et al. demonstrated a subpopulation with high ALDH activity in the osteosarcoma cell line OS99-1 [24]. They were able to grow xenografts in NOD/SCID mice with this cell line, and the cells with high ALDH activity isolated from the xenografts showed greater tumorgenicity, generating new tumors with as few as 100 cells. These ALDH-high cells showed characteristic cancer stem cell features of self-renewal, ability to produce differentiated progeny, and increased expression of the stem cell genes OCT3/4A, NANOG, and SOX-2.

Murase et al. used the approach of sorting with Hoechst dye to study 7 osteosarcoma cell lines (NY, OS2000, KIKU, Huo9, HOS, Saos2 and U2OS) and 1 bone malignant fibrous histiocytosis cell line (MFH2003) [25]. Of these, only the NY osteosarcoma cell line and the MFH2003 cell line showed a significant SP. Further testing of the MFH2003 cell line showed increased sphere-forming activity and soft agar colony formation in the SP cells. Implantation of serial dilutions of SP and nonSP cells into immunodeficient mice showed that 1 of 5 mice injected with 10^3^ SP cells grew tumor, while tumor growth was not seen in nonSP cells when fewer than 10^5^ cells were injected.

### 4.2. Ewing's Sarcoma

 Suvà et al. used CD133 to identify a subpopulation of Ewing's sarcoma cells that demonstrate tumor-initiating activity and sustained growth through serial xenotransplantations, reestablishing after every *in vivo* passage a cellular hierarchy of TICs (CD133+) and progeny (CD133−) [26]. Additionally, they found CD133+ Ewing's sarcoma cells capable of differentiation to adipogenic, osteogenic, and chondrogenic lineages. The CD133 cells also expressed significantly higher levels of the stem cell genes OCT4, SOX2, and NANOG. This same group later expressed the fusion gene, EWS-FLI1, which characterizes most Ewing's sarcomas, in pediatric mesenchymal stem cells, and demonstrated that it induced a transcriptome similar to that of Ewing's sarcoma family tumors [27]. These transformed cells also appeared to have a subpopulation that expressed CD133, the stem cell genes OCT4, SOX2, and NANOG, and *in vitro* sphere-forming ability. Prompted in part by these findings, Jiang et al. investigated the expression of CD133 in primary Ewing's tumors and cell lines to see if there was a correlation between CD133 expression and chemoresistance [28]. Of the 48 sources tested, most had very low or absent expression of the CD133− encoding gene PROM1 while 4 cases had overexpression of PROM1. Of these 4, 2 were found to have quickly developed a chemoresistant tumor while the other 2 were long-term survivors after receiving chemotherapy. These results suggest heterogeneity of CD133 expression in Ewing's sarcomas and a variable prognostic impact of the level of expression.

Awad et al. tested ALDH activity in Ewing's sarcoma [35]. Five Ewing's sarcoma cell lines were treated with ALDEFLUOR, and the 2% of cells with the highest ALDH activity (ALDH-high) were isolated as were the 2% of cells with the lowest ALDH activity (ALDH-low). Unsorted “flowthrough” cells were also collected as controls. The ALDH-high population in all five cell lines showed increased clonogenicity, sphere-forming ability, and 4 of the 5 cell lines showed ALDH-high cells to be more resistant to doxorubicin than ALDH-low and flowthrough cells. The fifth cell line, A4573, is known to be inherently resistant to doxorubicin [29]. Chemoresistance was hypothesized to be due to higher expression of ATP-binding cassette (ABC) transporter proteins causing drug efflux. There was significantly increased Hoechst dye efflux seen in ALDH-high cells as compared to ALDH-low cells. The ALDH-high cells were also able to produce a heterogeneous population of both ALDH-high and ALDH-low cells, while ALDH-low cells only produced ALDH-low progeny. ALDH-high cells expressed significantly higher levels of the stem cell genes OCT4, BMI-1, and NANOG. Finally, *in vivo* experiments were performed with two of the cell lines, TC71 and MHH. As few as 160 ALDH-high cells were able to generate a serially transplantable tumor in NOD/SCID/IL-2R*γ* null mice, while no tumor growth was seen with 80,000 or less ALDH-low cells, and 3 of 7 mice injected with 80,000 unsorted cells grew tumor, but none grew tumor with less than 80,000 unsorted cells injected. Immunohistochemistry showed ~1% of cells in xenografted tumors staining intensely for ALDH, including the tumors that grew from ALDH-high cell injection, suggesting the ability to generate heterogeneous progeny. Finally, 22 primary biopsy specimens from patients with Ewing's sarcoma were tested with immunohistochemistry and demonstrated a spectrum of ALDH expression with a small minority of the cells exhibiting very intense staining for ALDH.

### 4.3. Rhabdomyosarcoma

 Walter et al. explored TICs in embryonal rhabdomyosarcoma [30]. They used serial passages of rhabdomyosarcoma spheres to enrich for TICs and found upregulation of the stem cell genes OCT4, NANOG, c-Myc, SOX2, and PAX3. These enriched rhabdomyosarcoma spheres also demonstrated a 100-fold increase in tumorigenicity, causing tumor formation in xenografts with 10^4^ enriched sphere cells compared to 10^6^ of the nonenriched cells. CD133 gene and protein expression was found to be upregulated in the rhabdomyosarcoma sphere population as compared to adherent rhabdomyosarcoma cells. CD133+ and CD133− rhabdomyosarcoma cells were isolated and injected into immunodeficient NOD/SCID mice and the CD133+ cells showed tumor growth at lower numbers of injected cells, 10^2^ versus 10^6^, although only 1 mouse out of each cohort of 4 grew tumor at the lower dilutions of CD133+ cells, 10^2^ and 10^3^. The CD133+ cells showed adipogenic, osteogenic, and chondrogenic differentiation potential. CD133+ cells were also chemoresistant compared to CD133− cells, with significantly increased colony formation in agar after treatment with cisplatin and chlorambucil. Finally, 76 patient samples of embryonal rhabdomyosarcoma were stained for CD133. Patients whose tumor expressed a low or intermediate level of CD133 had an overall survival rate of 75%, while patients with high CD133-expressing tumors had a significantly lower overall survival rate of 50%.

### 4.4. Nonrhabdomyosarcoma Soft Tissue Sarcoma

 Terry and Nielsen have shown subpopulations of CD133-expressing cells in 5 of 5 primary synovial sarcomas and 3 of 3 synovial sarcoma cell lines [31]. However, to date, TIC properties were not assessed in these CD133+ cells compared to the rest of the tumor cells.

 Wu et al. examined 29 primary human tumors of mesenchymal origin including 7 aggressive fibromatosis, 5 osteosarcomas, 3 chondrosarcomas, 3 synovial sarcomas, 2 leiomyosarcoma, 4 malignant fibrous histiocytomas, 1 myxoid liposarcoma, 1 pleomorphic liposarcoma, 1 dermatofibrosarcoma protuberans, 1 myxoid chondrosarcoma, and 1 chordoma [32]. They identified a SP in all but 6 of the tumors tested, the dermatofibrosarcoma protuberans, the myxoid chondrosarcoma, 1 of the chondrosarcomas, 1 malignant fibrous histiocytoma, 1 leiomyosarcoma, and a synovial sarcoma. They observed a correlation between the size of the SP and tumor grade. SP and nonSP cells from one osteosarcoma, one synovial sarcoma, and 2 malignant fibrous histiocytosis samples were injected into NOD/SCID mice. The SP cells generated tumors at a higher frequency and with fewer injected cells than the nonSP cells. The SP-generated tumors were also significantly larger and heavier and were more readily transplantable. Finally, only tumors derived from the SP cells were able to repopulate both the SP and nonSP fractions when stained with Hoechst dye and resorted.

## 5. Conclusions

Identification of SICs is fraught with difficulties. Underlying many of the challenges in this field is the lack of accepted means by which to isolate normal mesenchymal stem cells. This issue clouds the ongoing debate about whether SICs reflect mesenchymal stem cells “gone bad” or if they arise from differentiated cells that have acquired stem-like properties as a result of the tumorigenic mutations [11]. The mesenchymal origin of some sarcomas even remains controversial, with some support, for example, for a neuroectodermal origin for these tumors [44]. Finally, sarcoma research struggles with the heterogeneity of the malignancies lumped under the term “sarcomas.” What holds true for an osteosarcoma may not be valid for a pleomorphic undifferentiated sarcoma.

Despite years of study, SIC research remains a fledgling field, building in large part on the findings from more common cancers such as breast, colon, and hematopoetic malignancies. Sheer numbers enable research in those cancers to progress more rapidly. Extrapolation from epithelial malignancies, though, may be misleading. One of the major concepts in the study of carcinoma TICs is the link between a stem-like phenotype and epithelial-to-mesenchymal transition (EMT). It is not clear how relevant EMT is to the development of SICs, which already have a mesenchymal phenotype.

 The methods employed to identify SICs share one important flaw: they are not able to isolate a pure TIC population. Each technique only selects for a population that is enriched for cells with stem-like properties. One could envision a Venn diagram of all the markers listed, and perhaps additional ones as they emerge, where the intersection of all markers would identify a pure SIC population. Some groups have evaluated the combination of CD133 and ALDH, for example, as a means of further purifying a TIC population in a variety of malignancies [45–48]. There are, however, many competing studies touting the superiority of one marker over another and few showing any additive or complementary effect of combining multiple markers, especially in sarcomas [44, 45]. Furthermore, whether the cells responsible for refractoriness to chemotherapy and those responsible for metastases are one, and the same has been called into question [1, 49]. It may be that one of the techniques for identifying TICs is more suited for identifying cells with metastatic potential while another technique identifies chemoresistant cells. Finally, Chaffer et al. have recently raised an additional confounding concept: a cancer cell might be able to convert to and from a stem-like state [50]. If “stemness” is a state that cells can adopt in response to environmental cues, for example, isolation of a pure TIC population may not be possible.

 The existence of TICs in sarcomas is an enticing proposition; it would explain in part why our success in treating these tumors has been limited. In addition, the existence of SICs would give us a target for new therapies that would complement existing treatments. Perhaps the means to identify these cells lies within the techniques and markers described above, or perhaps the next proposed marker will be the key. For this, we may have to continue to look towards the advances our colleagues are making in studying more common tumors. We must, however, also be judicious in adopting strategies that may not apply to our field. The identification of SICs would be greatly aided by a better understanding of the origin of sarcomas and TICs in general and establishment of clear criteria for the testing of proposed TIC subpopulations. If what we are looking for is not clearly defined, we will never find it.

## Figures and Tables

**Table 1:** Techniques used to isolate sarcoma-initiating cells.

Technique	Description
Surface markers (e.g., CD133)	Cells are incubated with fluorescent antibodies to specific surface markers. Flow-cytometry is used to isolate cells expressing surface marker

Aldehyde Dehydrogenase (ALDH)	Cells are incubated with reagent that is activated to fluorescent state by ALDH. Flow-cytometry is used to isolate the cells with the most ALDH activity

Side population analysis (dye exclusion)	Cells are incubated with a fluorescent dye such as Hoechst 33342. Flow-cytometry is used to select the population that excludes the dye, referred to as the “side population,” because of its location on the left-most portion of the flowplot

**Table 2:** Studies showing isolation of sarcoma-initiating cells.

Tumor Type	Technique	Summary of findings	Reference
Osteosarcoma		CD133+ cells demonstrated increased sphere formation, growth in soft agar, expression of OCT3/4, and presence of side population	
	CD133		[21, 22]
	
		Double positive (CD117+ and Stro-1+) cells were seen with a higher incidence in spheres, and they showed higher tumorigenicity as well as chemoresistance	
	CD117, Stro-1		[23]
	
		ALDH-high cells isolated from xenografts established from cell line OS99-1 had increased tumorigenicity, self-renewal, and ability to produce differentiated progeny, and expressed increased levels of OCT3/4A, NANOG, and SOX-2	
	
	ALDH		[24]
	
	
		SP was seen in 1 of 7 osteosarcoma cell lines tested. SP population had increased sphere- and colony-forming activity and increased tumorigenicity	
	SP		[25]
	

Ewing's sarcoma		CD133+ cells showed increased tumorigenicity, ability to establish a heterogeneous population and differentiation, and increased expression of OCT4, SOX2, and NANOG. There was a correlation in primary tumors between higher expression of CD133 and chemoresistance	
	
	CD133		[26–28]
	
	
		ALDH-high cells showed increased sphere- and colony-forming ability, chemoresistance, SP, and tumorigenicity	
	ALDH		[35]
	

Rhabdomyosarcoma		Serial passages of rhabdomyosarcoma spheres enriched for cells with increased expression of OCT4, NANOG, c-Myc, SOX2, and PAX3 and increased expression of CD133 and CD133+cells showed increased tumorigenicity, ability to Differentiate, and chemoresistance. CD133 expression also correlated with poorer survival in patient samples	
	
	
	CD133		[30]
	
	
	

Synovial sarcoma		5 of 5 primary tumor samples and 3 of 3 cell lines demonstrate a CD133+ subpopulation, but not TIC properties were tested	
	CD133		[31]
	

Multiple sarcomas		Size of SP in primary tumor samples correlated with tumor grade. SP cells from 1 osteosarcoma, 1 synovial sarcoma, and 2 malignant fibrous histiocytosis samples showed increased tumorigenicity and ability to produce a heterogeneous cell population (SP and nonSP)	
	
	SP		[32]
	
	
